# Occupational Traffic Accidents among Teachers in Spain

**DOI:** 10.3390/ijerph19095175

**Published:** 2022-04-24

**Authors:** Vicente Joaquín Delgado-Fernández, María del Carmen Rey-Merchán, Antonio López-Arquillos, Sang D. Choi

**Affiliations:** 1 Ph.D. Program Mechatronics Engineering, School of Industrial of Industrial Engineers, University of Málaga, 29071 Malaga, Spain; vizdlg@uma.es; 2 Consejería de Educacion y Deporte, 29071 Malaga, Spain; mmccrrmm@gmail.com; 3 Department of Economics and Business Management, School of Industrial Engineers, University of Málaga, 29071 Malaga, Spain; 4 Department of Occupational and Environmental Safety and Health, University of Wisconsin-Whitewater, Whitewater, WI 53190, USA; chois@uww.edu

**Keywords:** carpooling, car sharing, smart mobility

## Abstract

Occupational traffic accidents are a leading cause of injuries or deaths among workers. Teachers in Spain are especially concerned about the problem of commuting due to their particular labor conditions. Multiple work-related factors are associated with the risk and severity of occupational traffic-related motor vehicle crashes. The objective of this research is to analyze the influence of the variables associated with the severity of occupational traffic accidents among teachers in Spain. A logistic regression model was used for the current study. The odds ratio (OR) and confidence interval (CI) were calculated for the injured worker on a sample of 20,190 occupational traffic accidents suffered by teachers. The results showed that women, Spanish nationality, younger than 55 years, and those driving a car were more likely to suffer a light crash. In contrast, men, foreign nationalities, older than 55 years, and those riding a motorbike were more likely to suffer a serious crash. Based on these findings, motor vehicle safety training could be designed and adapted to the riskiest profiles. Additionally, effective mobility plans for commuting could help reduce work-related traffic accidents.

## 1. Introduction

Traffic accidents are a worldwide cause of safety concern [1]. Every day, more than 3500 people die in crashes involving vehicles, bikes, or pedestrians [2]. The economic consequences of fatal and nonfatal crash injuries from 2015 to 2030 are estimated to be USD 1.8 trillion worldwide [3]. Different causes are associated with traffic accidents. Some studies have reported the characteristics of the vehicles involved [4,5], the appropriate use of seat belts [6,7], drivers’ ages, [8,9], and human behavior [10,11]. It is important to highlight that some traffic accidents have been linked to work. In the group of accidents at work, traffic crashes caused a major amount of deaths [2]. Occupational traffic accidents can be classified as commuting accidents (accidents occurring during the travel to/from work, [12]) or on-duty traffic accidents. Depending on the country, commuting traffic accidents may or may not be officially considered as occupational accidents. In Spain, they are considered occupational accidents, while in countries such as the United Kingdom, they are only considered traffic accidents [13]. Different studies have found that drivers involved in traffic crashes while commuting were more likely to be severely injured.

The majority of workers and occupations are affected by the problem of commuting accidents [12], and the negative consequences of these accidents cannot be underestimated. Some studies have concluded that drivers involved in traffic crashes while commuting were more likely to be severely injured [14]. However, the number of studies focused on the factors associated with traffic accidents are limited. Additionally, in the particular case of professional drivers, they are exposed for a high percentage of their working time. It is worth mentioning that a previous study on the occupational risk of road accidents estimated that traffic accidents were 20–40% of occupational accidents with injuries in industrial countries [15]. In the USA, motor vehicle crashes are also the leading cause of work-related deaths. From 2003 to 2018, more than 29,000 workers in the USA died in a work-related motor vehicle crash [16]. In 2019, 1270 U.S. workers driving or riding in a motor vehicle on a public road died in a work-related crash (24% of all work-related deaths) [17].

Teachers in Spain are especially concerned about the problem of commuting accidents [18]. In Spain, more than 800,000 teachers work in primary and secondary education. Due to their particular labor conditions in public education, they may have to change workplaces each academic year. As a consequence, many of them have to travel long distances in their daily commutes. As a result, they suffer multiple occupational injuries associated with traffic accidents [18]. Although there are no official data on the exposure time of teachers to the traffic risks, data from previous studies pointed out that traffic accidents caused the majority of occupational deaths in the sector [18].

Occupational injuries suffered by teachers have been previously studied in the literature [19]. Some authors identified risk factors for repetitive strain injuries [20], gender differences in occupational accidents [21], and injury trends related to frequency and severity in urban schools [22]. Other researchers found that working conditions such as stress, job demands, and poor sleep quality were associated with musculoskeletal pain in public school teachers [19,23]. Despite the previous research that analyzed teachers’ occupational injuries, more specific studies focused on occupational traffic accidents among teachers are warranted. Thus, the objective of this research is to analyze the influence of the variables associated with the severity of occupational traffic accidents among teachers in Spain.

## 2. Materials and Methods

### 2.1. Data Collection

Every occupational accident that occurs in Spain should be reported to the Spanish labor authority using the electronic system Delt@ to submit an official accident report. As a result, the data provided by the system are a significant sample of the occupational accidents that occurred in the country. Previous studies have used accident data from the Delt@ system to study the variables of occupational accidents, in general [24], and occupational crashes, in particular [12,25].

In total, 102,778 occupational accidents among teachers from 2009 to 2019 in Spain were provided, and 20.5% of them (21,090) were reported as occupational traffic accidents. The majority of crashes (98.5%) were considered light accidents, and only 0.1% had fatal consequences.

**Figure 1:**
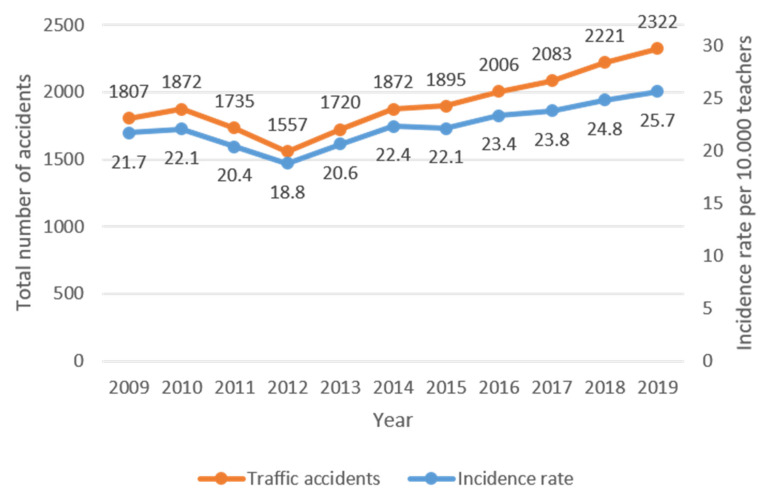
Traffic accidents and incident rates of teachers by year.

It is important to note that, in Spanish legislation, commuting accidents were considered occupational accidents. They represented 84.5% of the sample analyzed. Women suffered 71.1% of crashes, while men suffered 28.9%. The majority of accidents (96%) were suffered by a single worker, and only 4% of accidents involved more than one worker. Incidence rates of teachers and traffic accidents were calculated and are shown in Figure 1.

### 2.2. Statistical Analysis

A logistic regression model was used for the current study. In this statistical model, the odds ratio (OR) was calculated for the injured worker with a sample of workers who have been exposed to some variable compared to another sample of workers not exposed. OR is defined as the excess or defect of advantage (“odds”) that the individuals exposed to the disease or condition have of presenting it versus not presenting it, with respect to the advantage of individuals unexposed to the condition presenting versus not presenting it [26]. The cited method is indicated when you want to know how a series of factors influence a response that is measured as a dichotomous categorical variable [27]. Statistical analysis was carried out using the Software Statistical Package for Social Science (SPSS, version 25). Based on the calculated ORs with a 95% confidence interval (CI), the relation between the severity of occupational traffic accidents and different variables of workers affected were analyzed.

## 3. Results and Discussion

Figure 1 shows that the number of traffic accidents suffered by teachers increased year by year. Although the total number of teachers also rose, the increase in the total number of accidents was higher.

Traffic accidents were classified according to their severity code, based on medical criteria (light, serious, very serious, or fatal). The distribution of the accidents was grouped according to the different variables included in the official accident report.

**Figure 2:**
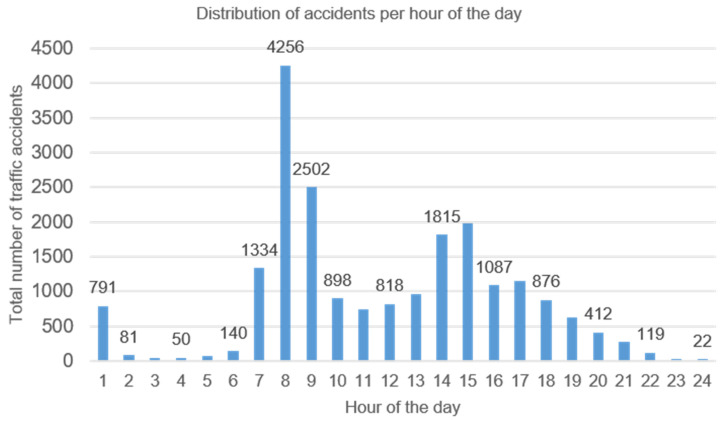
Distribution of total number of accidents analyzed, according to the hour of the day.

In Figure 2, the distribution of the total number of occupational crashes notified by teachers from 2009 to 2019, according to the hour of the accident, is shown. The majority of accidents were concentrated at the beginning of the working journey (8 a.m. to 9 a.m.) and the end of the school journey (2 p.m. to 3 p.m.). Accidents at the end of the journey were distributed in different hours due to the staggered exit time of teachers, caused by the extracurricular activities of students.

**Figure 3:**
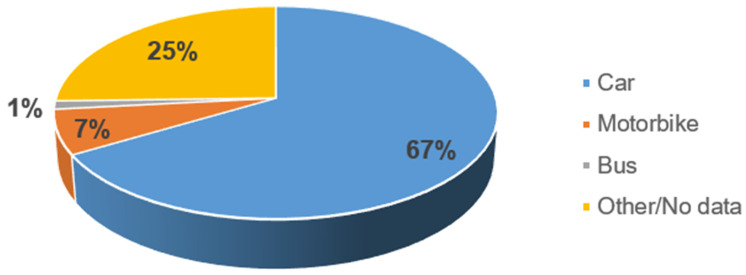
Distribution of teachers’ accidents per vehicle involved.

In addition, occupational traffic accidents notified by teachers were classified according to the vehicle or transportation involved in the crash. As shown in Figure 3, the majority of accidents involved cars and motorbikes.

The results obtained in the calculation of the OR and CI are shown in Table 1. The most significant values are described in the following subsections.

### 3.1. Gender

The number of female teachers in Spain has increased in the last few years. Women represented 66% of teachers in the past decade; however, the percentage of injured teachers in occupational traffic accidents in the same period was higher (71.1%). The sex variable was significantly and independently associated with the severity of occupational traffic accidents. An injured male teacher was less likely to suffer a light accident (OR = 0.36; CI, 0.2–0.4) than a female. An injured male teacher was more likely to suffer a fatal traffic accident than a female (OR = 3.13; CI, 1.4–6.9). These results are in accordance with previous research on traffic accidents that found greater mortality rates among males. In a study developed in France, the results showed that the male/female incidence rate was 3.1 for mortality (95% CI: 3.0–3.3) [28]. Then, teachers’ occupational crashes did not show differences in terms of sex when they were compared with global results of traffic accidents. The sex differences in fatal traffic accidents can be justified by their lower perception of risks [29,30], lower use of vehicle safety devices such as seatbelts [31,32], and risky behaviors [33,34]. Despite the fact that accidents suffered by women had lower severity, they were more likely to suffer commuting accidents than males [25].

Other habitual injuries suffered by teachers such as neck or multiple injuries were associated with a worse OR for female teachers than male teachers [18].

### 3.2. Type of Contract

Temporary workers were associated with worse accidents rates and more severe injuries from occupational accidents. In the case of occupational crashes, the victims were more often on permanents contracts [15]. However, in the specific group of occupational traffic accidents suffered by teachers, the OR values were not significant, because all CI were included in the unit. It was not possible to analyze significant results based on the type of contract of the teachers injured.

### 3.3. Risk Assessment

Risk assessment is a legal requirement in Spain for any company or organization with workers. Despite this legal obligation, a high percentage of organizations do not have a risk assessment [35]. An injured teacher working in an organization with a risk assessment presented a lower risk of light severity (OR = 0.69, CI, 0.5–0.8) than another working in an organization without risk assessment. For the rest of the severities studied, the results obtained were not statistically significant.

### 3.4. Nationality

With regard to the nationality of the teachers injured, some significant differences were found. Injured Spanish teachers were more likely to suffer a light occupational traffic accident (OR = 1.7, CI, 1.1–2.8) than foreign teachers. On the other hand, a Spanish teacher was less likely to suffer a serious crash (OR = 0.53, CI, 0.3–0.9) than a foreign teacher. Similar results (OR = 1.6 for Spanish workers and light accidents) were obtained in previous research [36]. Aligned with the cited results, more studies associated the nationality and severity of crashes [37]. These results can be motivated by several reasons, such as unsafe traffic behavior and differences in the safety education of migrant workers [38].

In contrast, the nationality factor was not found to be significant in some habitual injuries of teachers such as back, lower extremities, or multiple injuries [18].

### 3.5. Age

Attending to the age of the injured teachers, the effect of age was found significantly and independently associated with the severity of the accidents analyzed. Traffic accidents among teachers older than 55 years old were less likely to be light (OR = 0.65, CI, 0.4–0.9), and the same group of workers presented higher OR for serious accidents (OR = 1.73, CI, 1.1–2.5). Similarly, an increase in the severity of injury produced in a traffic accident among older workers has also been previously documented [39,40,41]. Aligned with the cited results, older teachers were the most prevalent group of workers affected by MSD caused by their habitual tasks [42,43].

### 3.6. Vehicle

Another important risk factor in traffic accidents identified in the literature is the vehicle involved [44,45]. For the light accidents analyzed, driving a car was considered a risk factor (OR = 1.95, CI, 1.5–2.4), while riding a motorbike (OR = 0.44, CI, 0.2–1.4) was a protective factor for light occupational traffic accidents among teachers. In contrast, riding a motorbike was identified as a risk factor for serious accidents (OR = 2.39, CI, 1.7–3.3), while driving a car was identified as a protective factor (OR = 0.48, CI, 0.3–0.6).

## 4. Conclusions

This research showed that personal and organizational factors were associated with the severity of occupational traffic accidents among teachers. Men and women presented different results. Female teachers had a disproportionately higher number of light traffic injuries, whereas male teachers were more involved in more severe or fatal traffic accidents. Older teachers were detected as a risky profile in the case of serious accidents. Similarly, the nationality of the worker and an updated risk assessment in the working site were detected as organizational factors related to the severity of injuries caused by crashes.

In contrast, other variables studied, such as the length of service, type of contract, or public/private schools, did not obtain results with statistical significance. It is remarkable that the fatal crashes did not show significant results, but this might be due to the low number of fatal crashes registered in the education sector.

Results from the current study can help reduce traffic accidents by designing preventive strategies adapted to the teachers’ profiles and their organizations.

Some policy interventions could reduce the number of traffic accidents suffered by teachers [46]. Due to the particularities of the education sector, mobility plans adapted to the teachers’ working conditions and integrated into the Occupational Health and Safety Management of the Schools should be promoted by regional governments and institutions. Currently the majority of road safety education programs are focused on children and high school students [47,48,49], but they are not adapted to the teachers’ profiles. Another positive intervention could be the promotion of institutional carpooling systems among teachers to reduce risky behaviors. In this sense, some authors pointed to the safer behavior of drivers during carpooling in terms of speeding and mobile phone use while driving [50].

### 4.1. Limitation of the Study

Although the variables analyzed were statistically significant, and many of them were considered relevant to the risk and the severity of the occupational crashes suffered by teachers, some additional data could improve the results obtained.

Data provided were collected through the official occupational accident form. As the design of this accident report was not focused on traffic accidents, some relevant variables pointed out in the literature such as road conditions, environment, or vehicle conditions [51,52] were not collected.

Similarly, the exposure time was associated with the risk of accidents in previous studies [7,15]; however, for the current study, it was not possible to obtain data about the exposure time of teachers during their commuting in a habitual working day. Hence, it was not possible to analyze the influence of cited variable.

### 4.2. Future Research

Additional information about the circumstances of the accidents should improve the results of the analysis. Occupational traffic reports supplied by National Traffic Authorities will be very useful to extend the current analysis and results.

A mobility survey focused on occupational displacements of the teachers could provide important information about the lack of data detected, such as exposure time to the traffic risks, type of road, number of passengers, and vehicle characteristics and conditions. The influence of psychosocial factors on the risk of occupational traffic accidents should be studied in future research. Some working conditions such as technostress, mental fatigue, or inadequate workload could impact negatively the risk of accidents in some sensitive workers’ profiles.

## Figures and Tables

**Table 1:** Odds ratio calculated for occupational traffic accident variables.

Odds Ratio	Light	IC	Serious	IC	V. Serious	IC	Fatal	IC
Men	0.36	0.2–0.4	2.79	2.1–3.5	1.56	0.6–4.0	3.13	1.4–6.9
Women	1		1		1		1	
Permanent	1.07	0.8–1.3	0.94	0.7–1.1	1.36	0.5–3.5	0.58	0.2–1.2
Temporary	1		1		1		1	
Risk Assessment	0.69	0.5–0.8	1.5	0.9–1.9	1.95	0.6–5.4	0.69	0.3–1.5
No Risk Assessment	1		1		1		1	
Spanish	1.7	1.1–2.8	0.53	0.3–0.9	0.99	0.9–1.0	0.75	0.1–5.6
Foreign	1		1		1		1	
older than 55	0.65	0.4–0.9	1.73	1.1–2.5	0.88	0.1–6.6	1	1.0–1.0
younger than 55	1		1		1		1	
Car	1.95	1.5–2.4	0.48	0.3–0.6	0.5	0.1–1.2	0.89	0.3–2.0
Other	1		1		1		1	
Motorbike	0.44	0.3–0.6	2.39	1.7–3.3	2.75	0.7–9.5	0.57	0.7–4.2
Other	1		1		1		1	
Bus	0.65	0.2–1.4	1.46	0.6–3.5	4.61	0.6–34.8	1	1.0–1.0
Other	1		1		1		1	

## Abbreviations

The following abbreviations are used in this manuscript: OR: Odds Ratio; CI: Confidence interval.
